# Supplementary material for: No evidence for a bovine mastitis Escherichia coli pathotype
Source: BMC Genomics. 2017 May 8;18:359. doi: 10.1186/s12864-017-3739-x (PMC5422975; doi:10.1186/s12864-017-3739-x)
Supplement: Supplementary file 3 — Minimum spanning tree (MST) of the MLST results. Figure S2. Phylograms and tanglegrams for the 25 bovine-associated E. coli genomes based on WGA core genome, gene and VF content. Figure S3. Manhattan plots of Fisher’s exact test p-values for the OG/VF pathotype (MAEC/commensal isolates) and phylogroup (A/B1) associations. Figure S4. Gene organization of the AMR-SSuT/Tn10 gene cluster. Figure S5. Circular genome diagrams for the MAEC 1303 and ECC-1470 replicons with GI and prophage positions. Figure S6. Heatmap of VF presence/absence, including gene names/locus tags. Figure S7. Gene organization of the Flag-2 and ECC-1470 T6SS/1 gene clusters. (PDF 18 Mb) [file 12864_2017_3739_MOESM3_ESM.pdf]

Figure S1

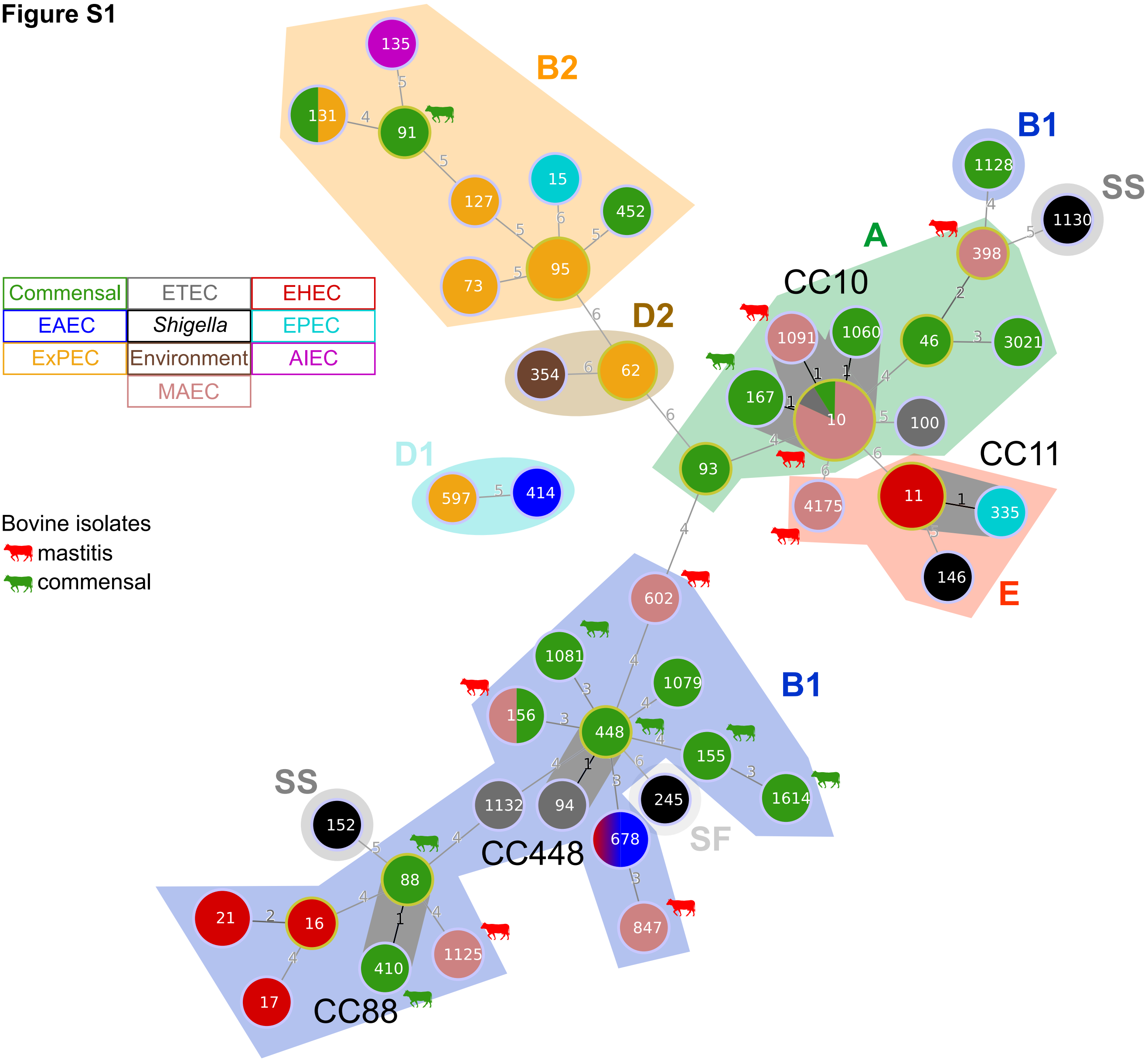

Figure S1. Minimum spanning tree (MST) of the multi-locus sequence typing (MLST) results generated with the goeBURST algorithm and visualized with PHYLOViZ. Sequence types (STs) are depicted by circles (nodes) with the respective ST number. The size of each ST node is proportional to the number of isolates allocated to the ST. Additionally, ST nodes are colored pie charts indicating the proportion of included pathotypes. Number of variants between each ST node are indicated on the edges. Clonal complexes (CCs) were designated for STs differing only by a single locus/allele (single locus variant, SLV). The STs belonging to a CC are highlighted by gray vertices and the CCs are named by the corresponding founder ST. Colored cows indicate STs with MAEC and commensal isolates. ECOR phylogenetic groups are highlighted by colored fields.

Figure S2 A

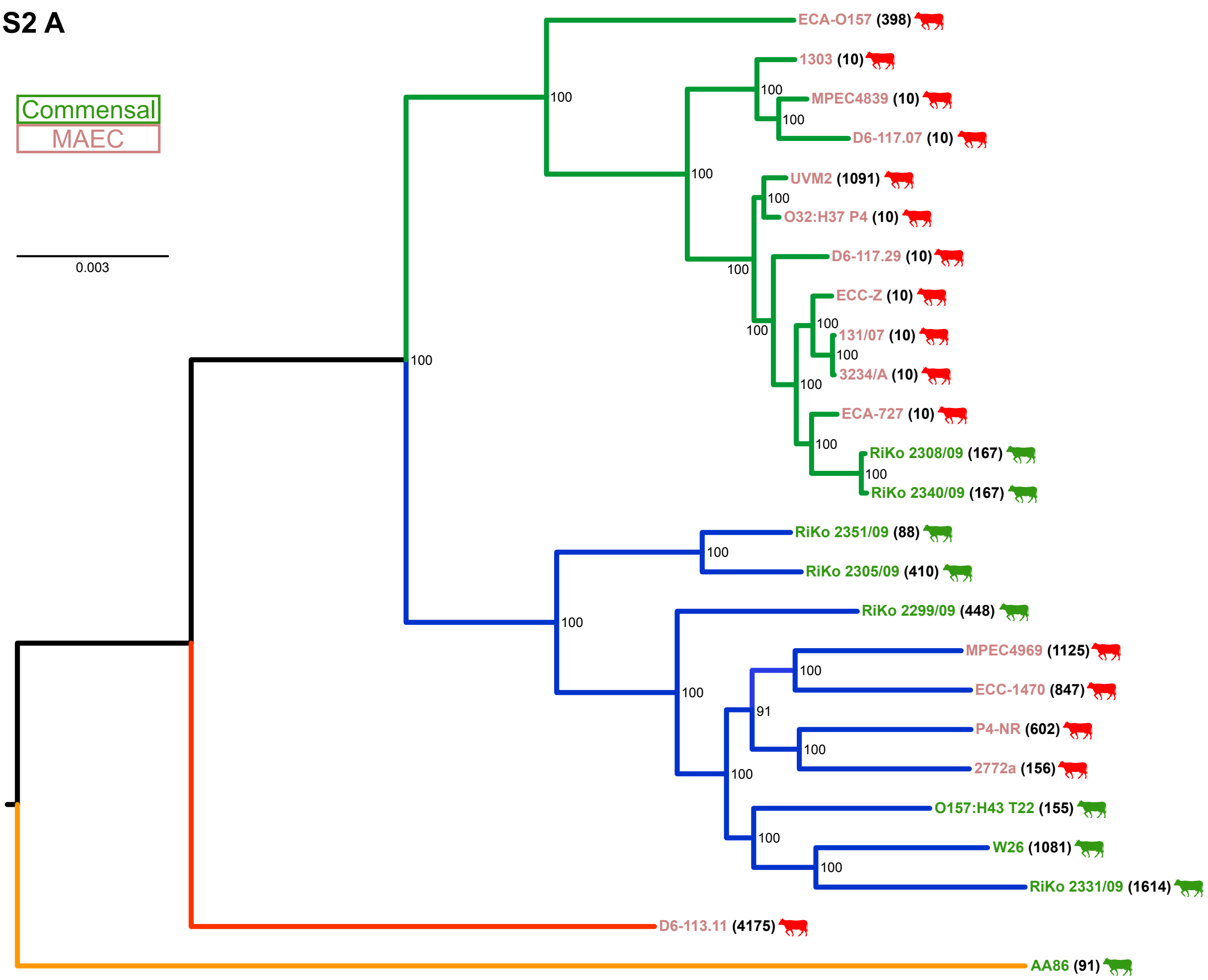

Figure S2 B

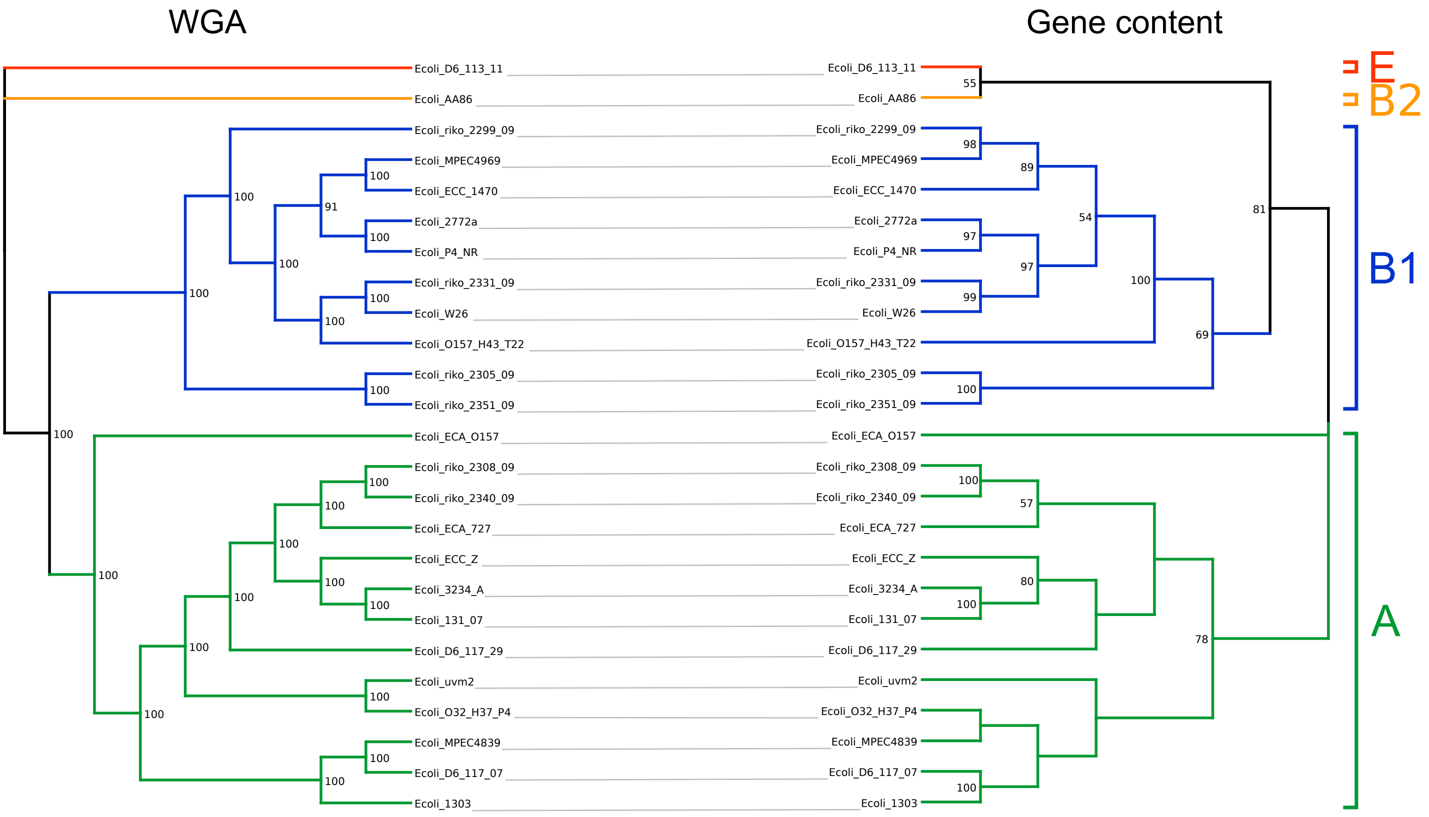

Figure S2 C

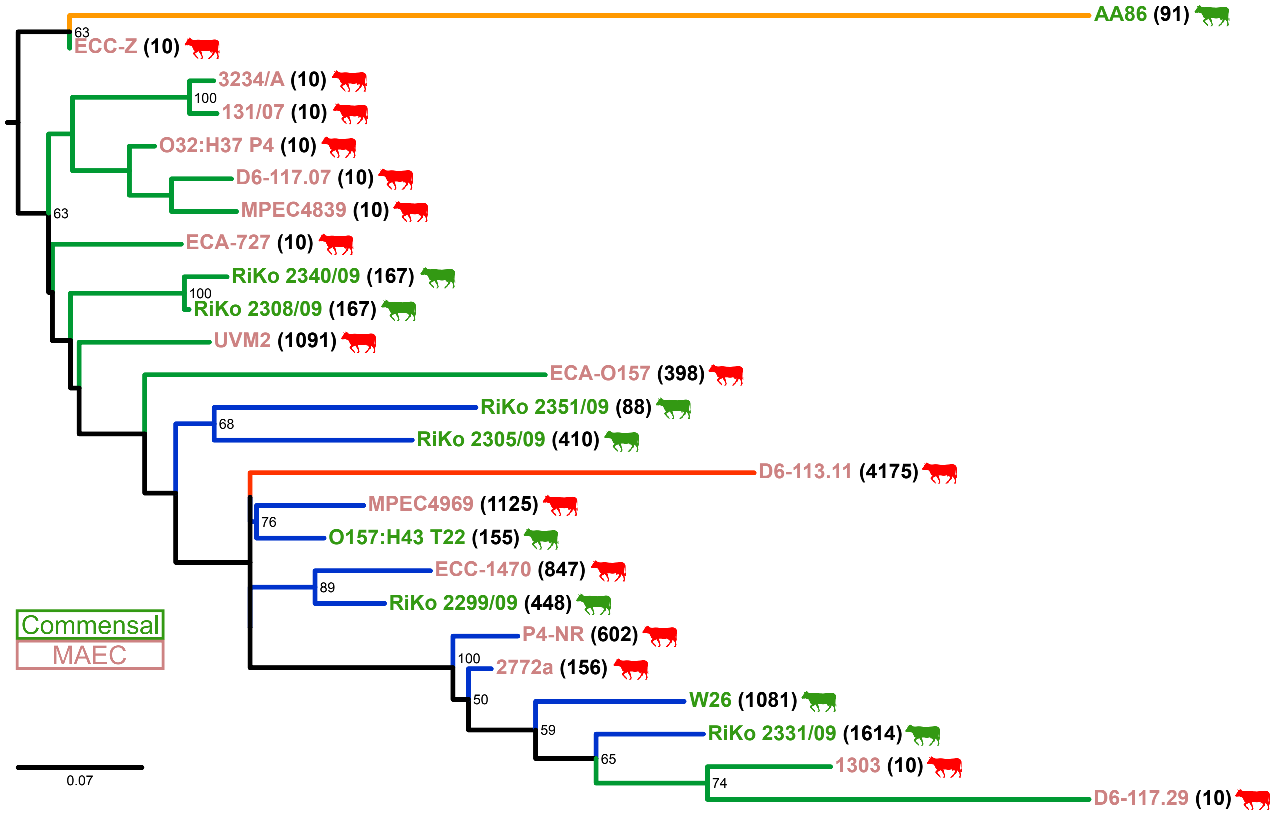

Figure S2 D

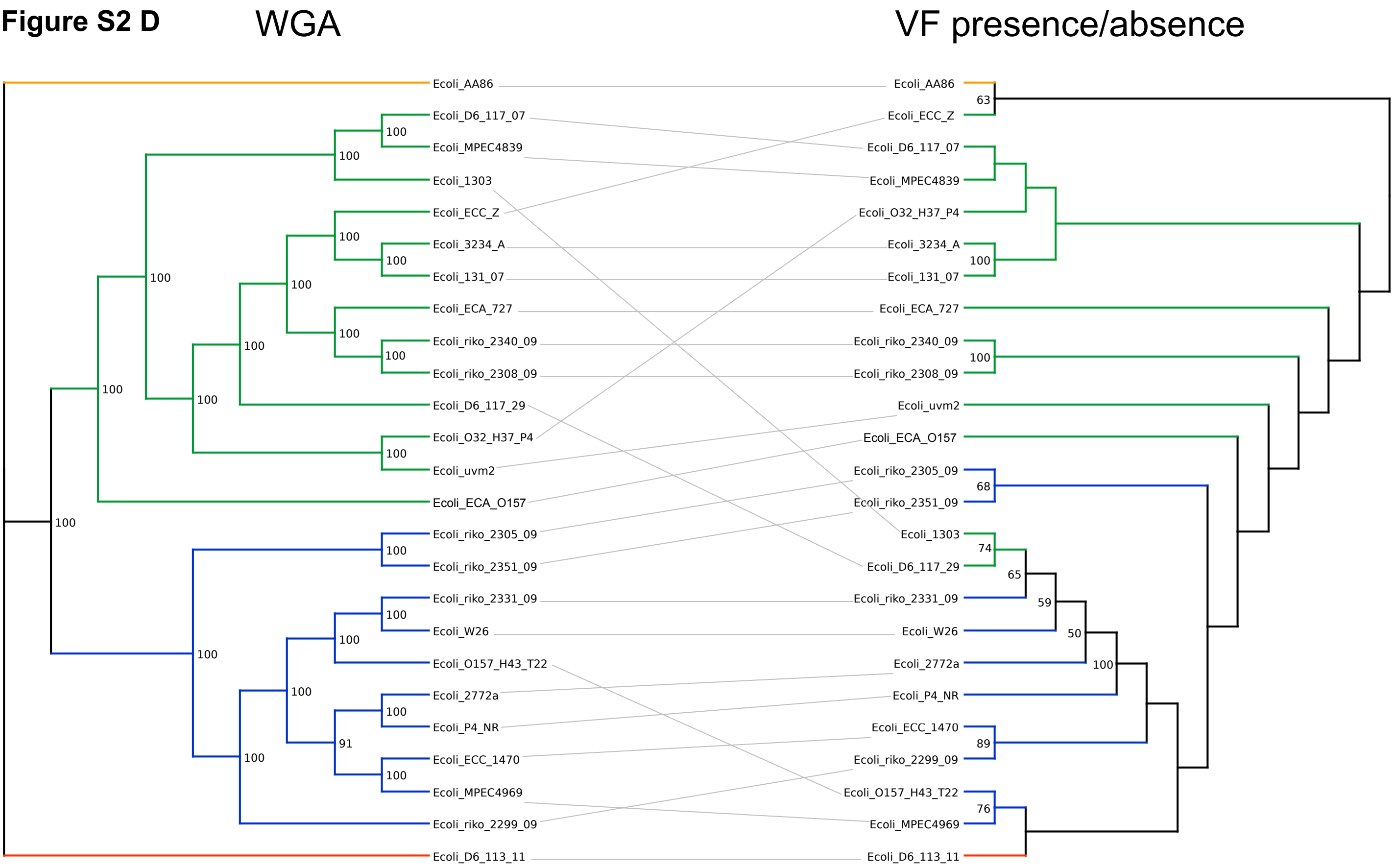

Figure S2. Phylograms and tanglegrams for the 25 bovine-associated *E. coli* genomes. ECOR phylogenetic groups are indicated by color: A (green), B1 (blue), E (red), and B2 (orange). The ST for each strain is given in parentheses. Bootstrap values below 50 were removed from all trees. **(A)** Whole genome alignment (WGA) phylogeny. The best scoring maximum likelihood (ML) phylogeny was inferred with RAxML's GTRGAMMA model from the core alignment length of 3,393,864 bp with 1,000 bootstrap resamplings. The tree was visualized with FigTree and midpoint rooted. **(B)** Tanglegram between the WGA genealogy and the gene content tree generated with Dendroscope. **(C)** Best scoring ML dendrogram (RAxML BINGAMMA) based on the presence/absence of 556 virulence-associated genes, with 1000 resamplings for bootstrap support values. The tree was visualized with FigTree and midpoint rooted. **(D)** Tanglegram between the WGA genealogy and the VF content tree generated with Dendroscope.

Figure S3 A

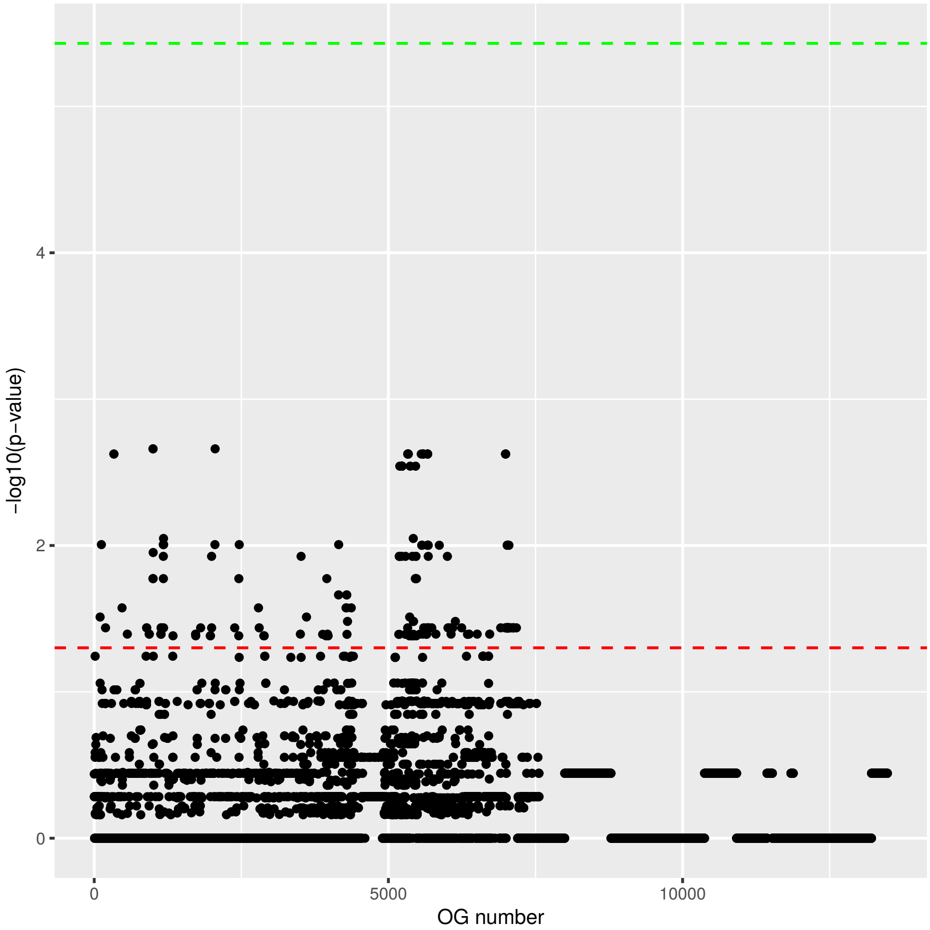

Figure S3 B

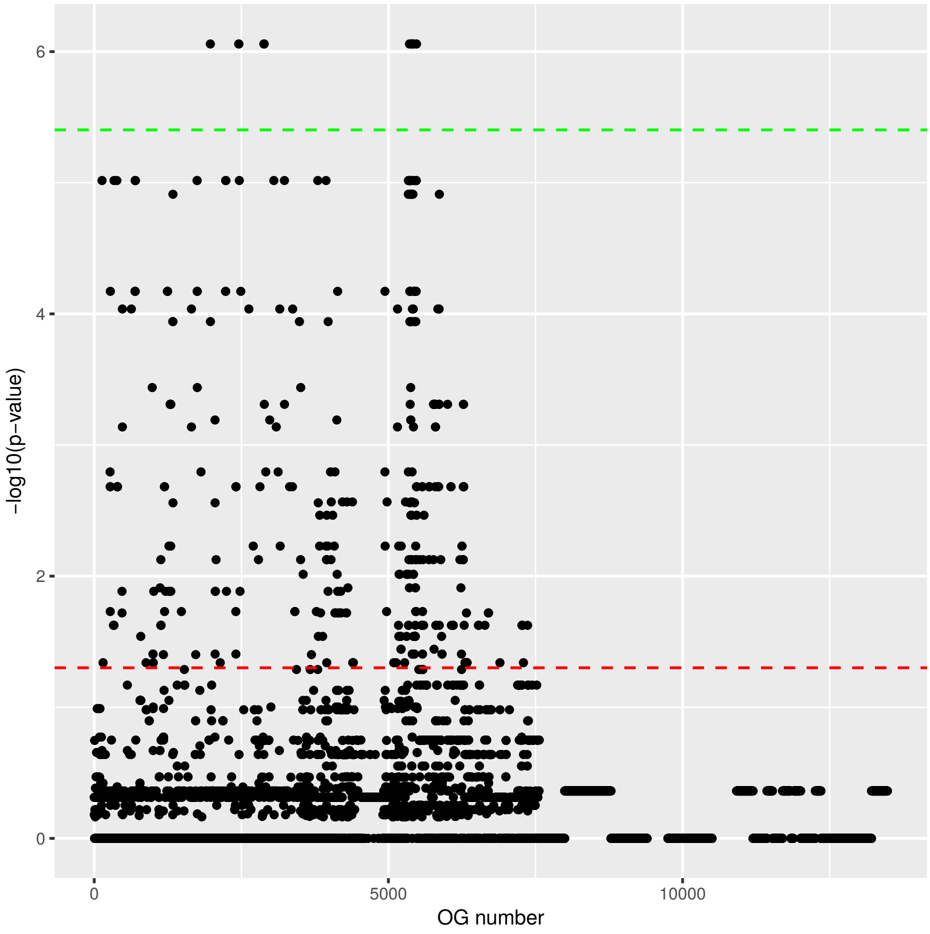

Figure S3 C

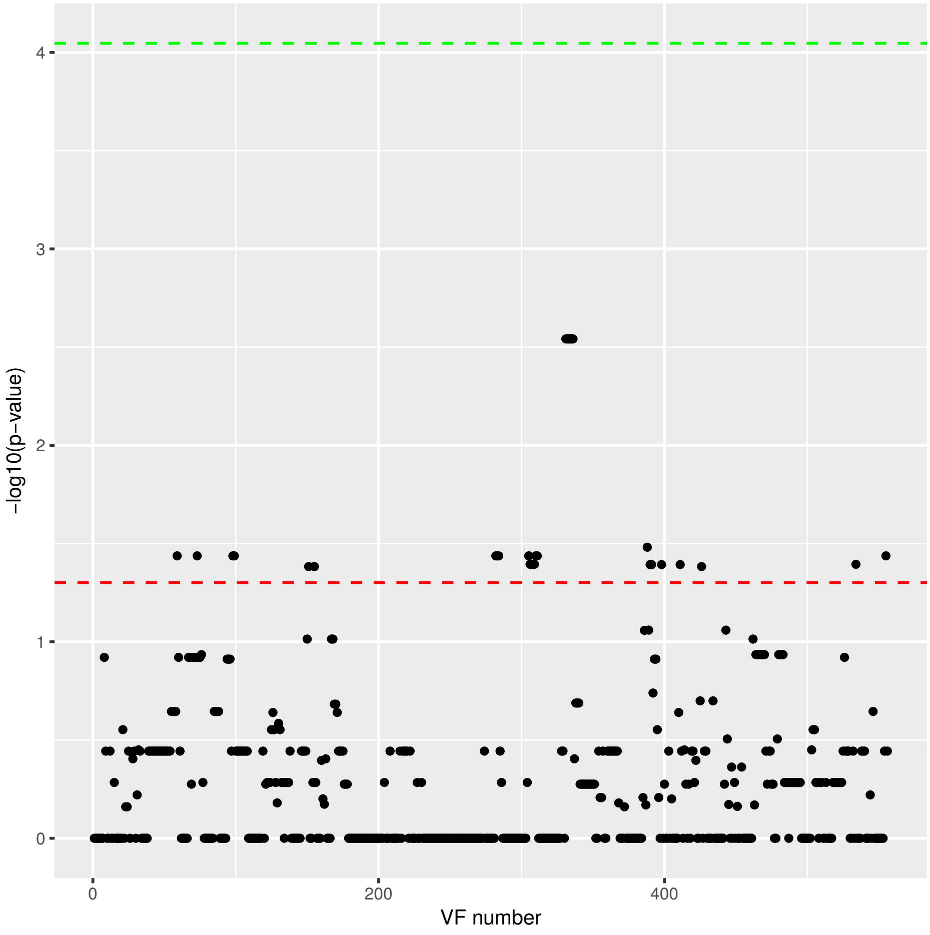

Figure S3 D

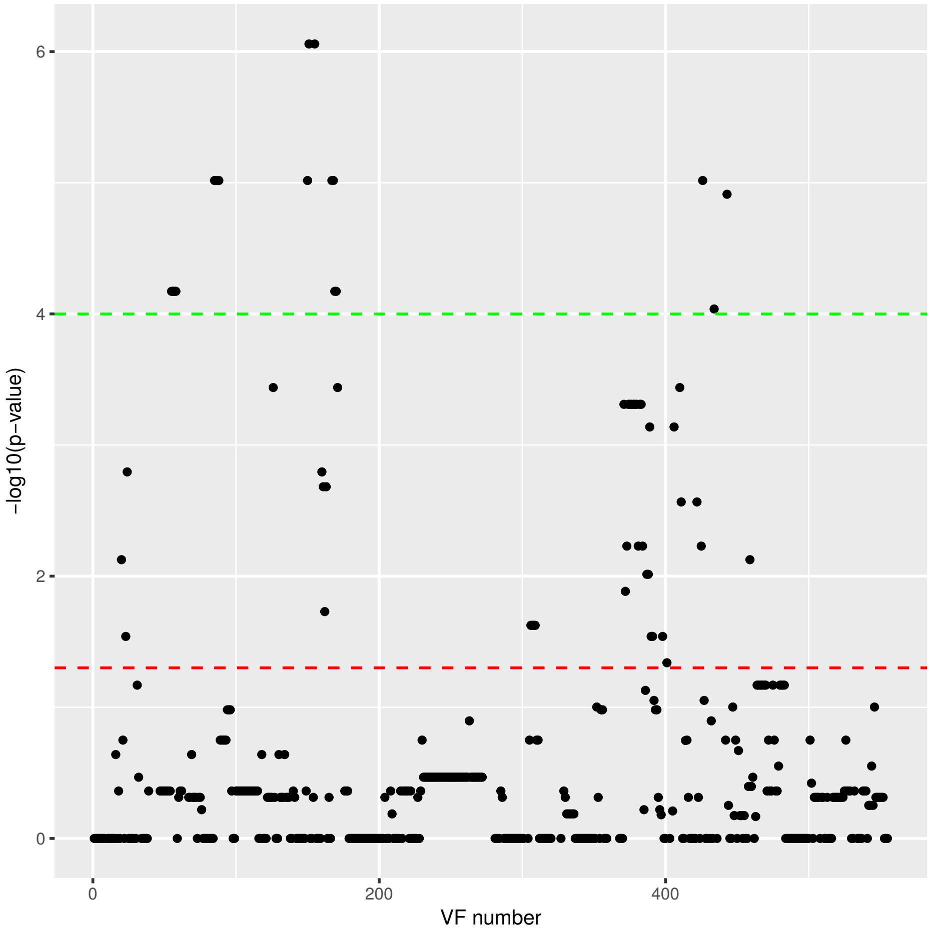

Figure S3. Manhattan plots of Fisher exact p-values for orthologous group (OG) or virulence factor (VF) associations of the 25 bovine-associated *E. coli* genomes. The red dotted line indicates the significance threshold (0.05), the green line the Bonferroni-corrected threshold. **(A)** OG association with pathotype (MAEC, commensal isolates) or **(B)** phylogroup (A, B1). **(C)** VF association with pathotype (MAEC, commensal) or **(D)** phylogroup (A, B1).

Figure S4

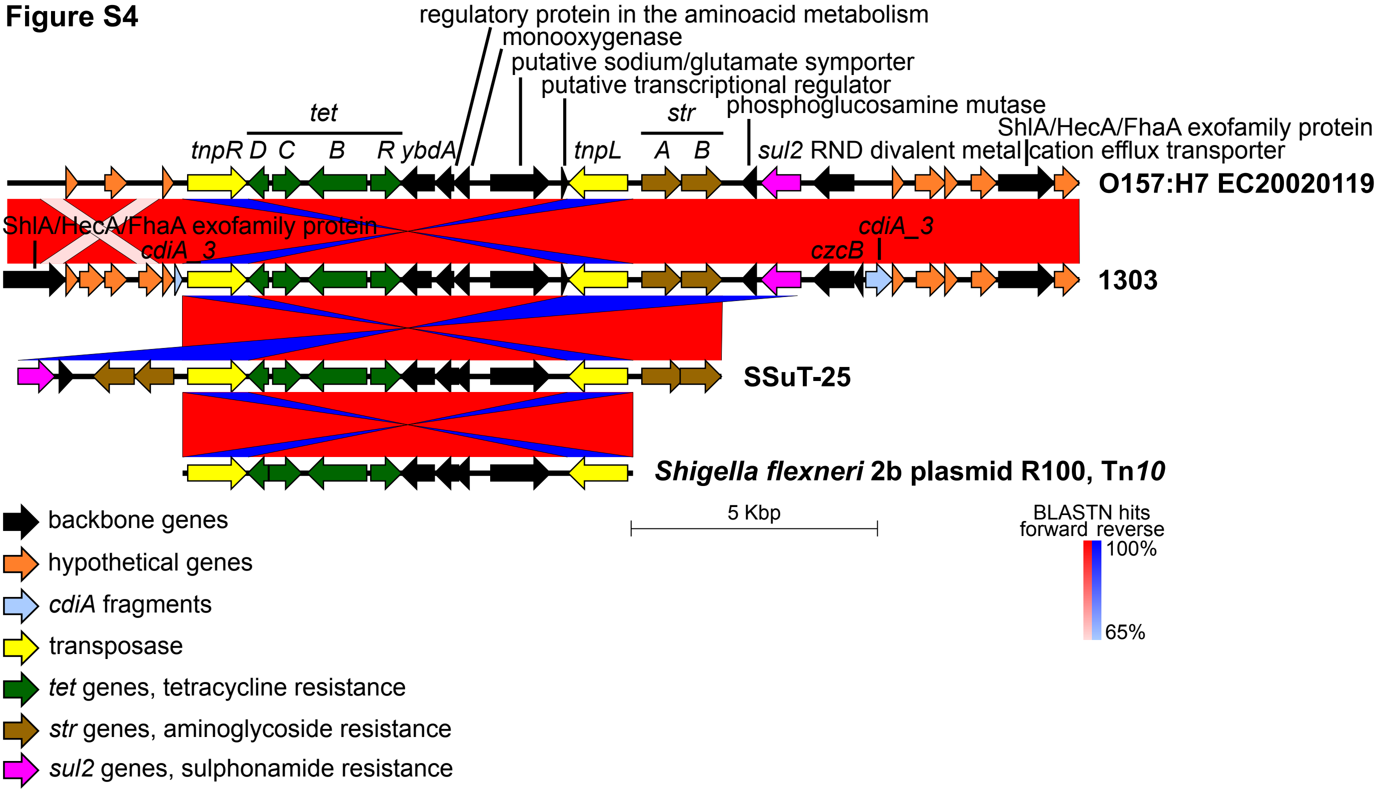

Figure S4. Gene organization of the AMR-SSuT/Tn*10* gene cluster. The comparison was done with the AMR-SSuT entities in strains MAEC 1303 (encoded on GI4), *E. coli* O157:H7 strain EC20020119, and *E. coli* strain SSuT-25, as well as Tn*10* of *Shigella flexneri* 2b plasmid R100. The diagram was created with Easyfig utilizing BLASTN+. Homologous regions are connected via red vertices for forward and blue vertices for inverted regions, and colored by nucleotide identity. Gene names are indicated above genomes encoding for these. Genes are colored according to their functions.

Figure S5 A

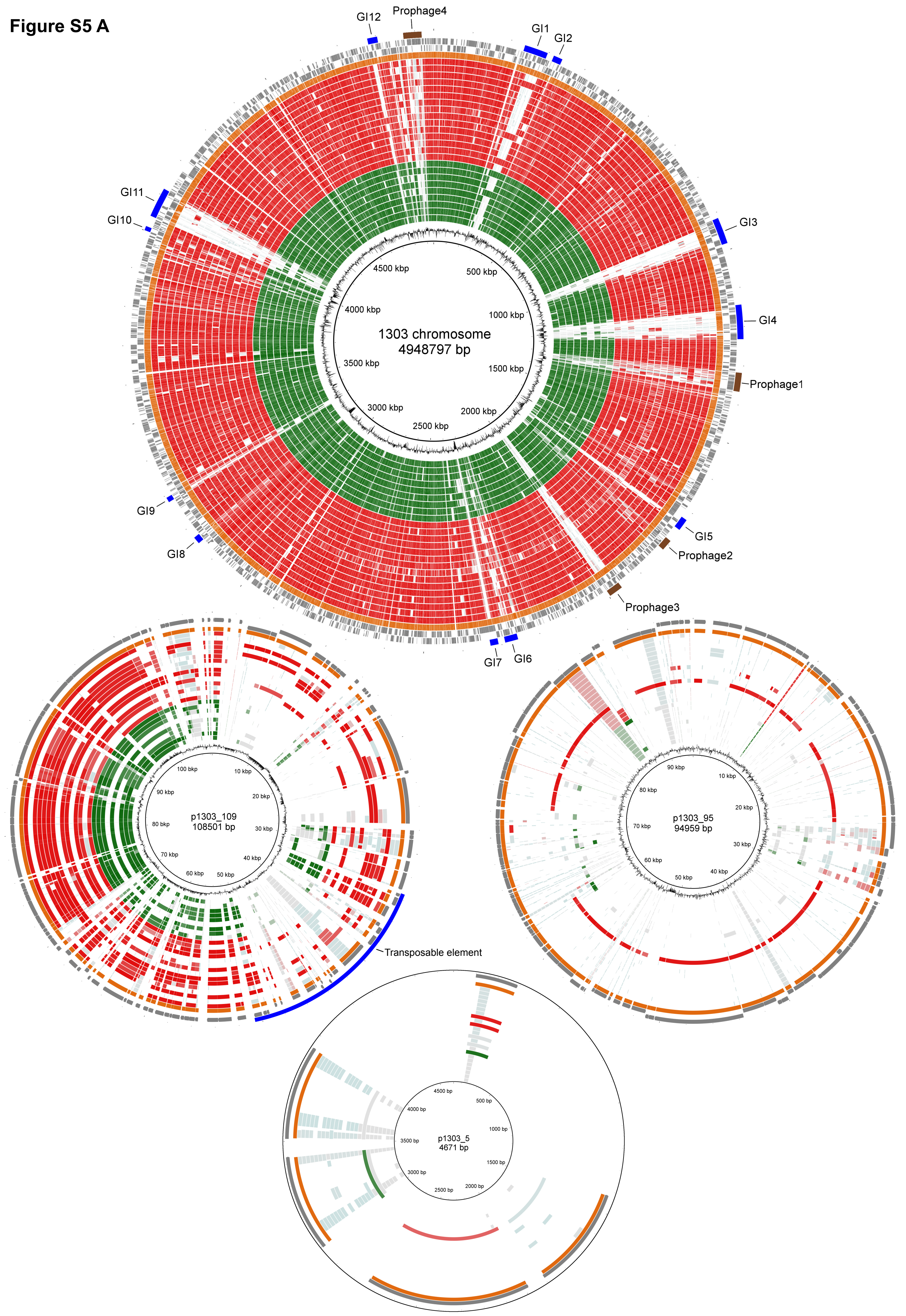

Prophage4
GI12
GI1
GI2
GI3
GI4
Prophage1
GI5
Prophage2
Prophage3
GI6
GI7
GI8
GI9
GI10
GI11
1303 chromosome
4948797 bp
500 kbp
1000 kbp
1500 kbp
2000 kbp
2500 kbp
3000 kbp
3500 kbp
4000 kbp
4500 kbp
p1303_109
108501 bp
10 kbp
20 bkp
30 kbp
40 kbp
50 kbp
60 bkp
70 kbp
80 bkp
90 kbp
100 bkp
Transposable element
p1303_95
94959 bp
10 kbp
20 kbp
30 kbp
40 kbp
50 kbp
60 kbp
70 kbp
80 kbp
90 kbp
p1303_5
4671 bp
500 bp
1000 bp
1500 bp
2000 bp
2500 bp
3000 bp
3500 bp
4000 bp
4500 bp

Figure S5 B

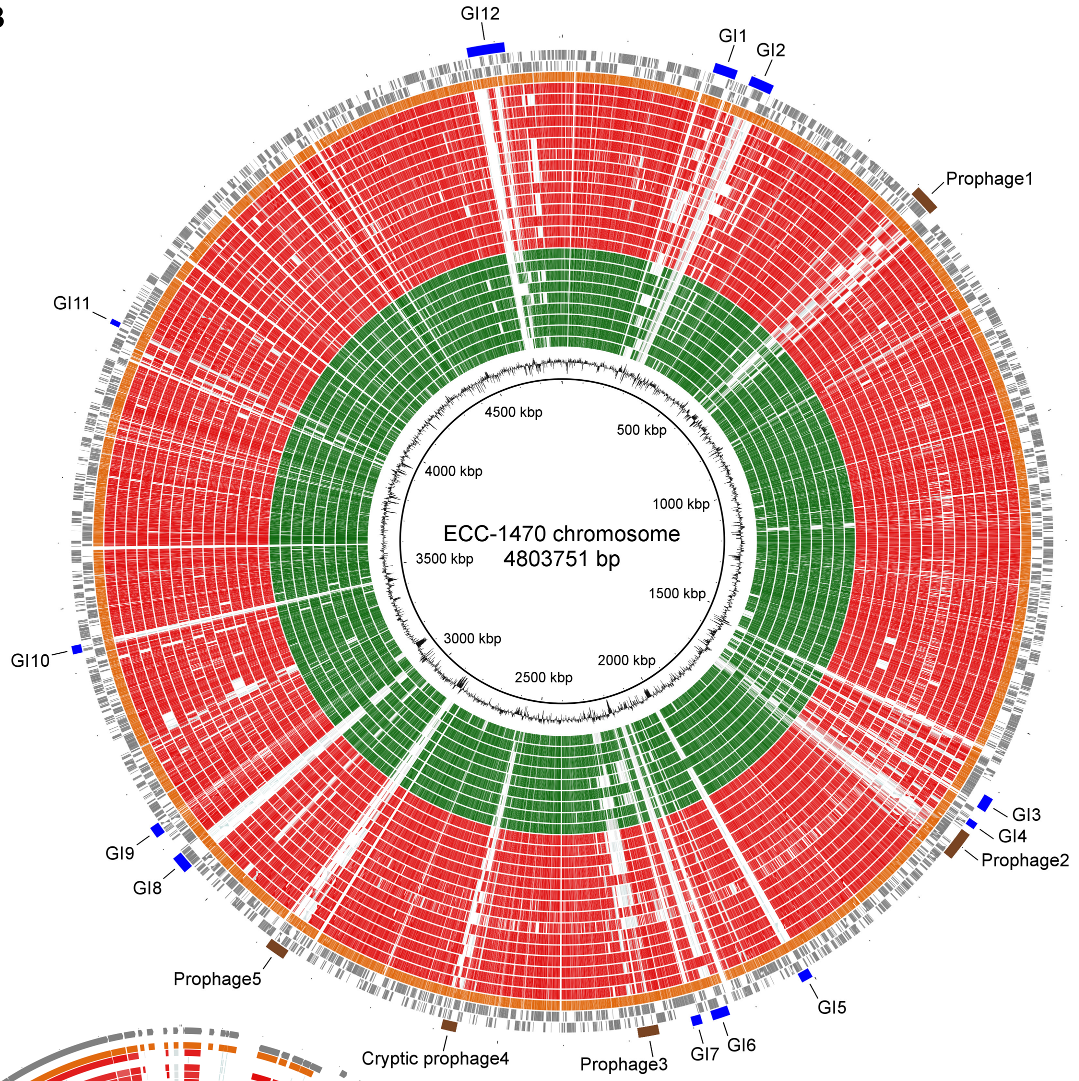

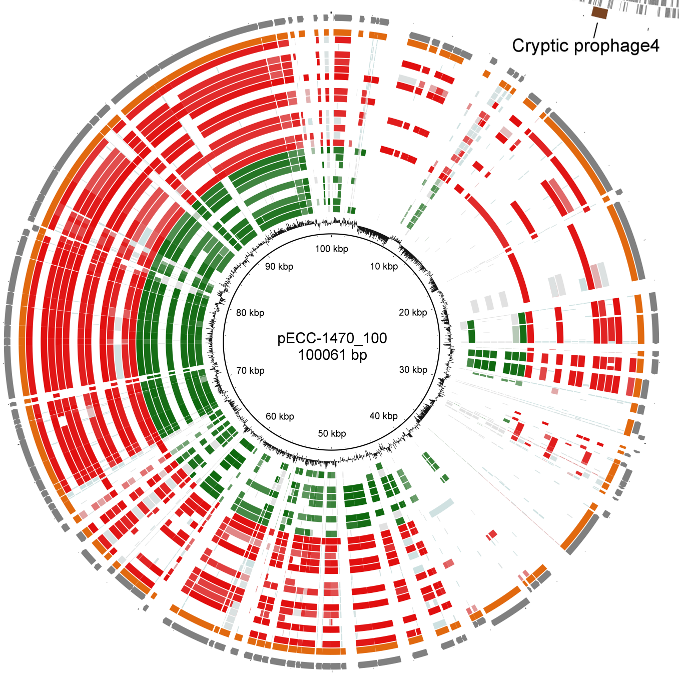

Figure S5. Circular genome diagrams created with BRIG. All figures have the following ring order (from inner to outer). **Black:** GC content. **Green** commensal strains: AA86, O157:H43 T22, RiKo 2299/09, RiKo 2305/09, RiKo 2308/09, RiKo 2331/09, RiKo 2340/09, RiKo 2351/09, and W26. **Red** MAEC: (1303 only in the ECC-1470 diagrams), 131/07, 2772a, 3234/A, D6-113.11, D6-117.07, D6-117.29, ECA-727, ECA-O157, (ECC-1470 only in the 1303 diagrams), ECC-Z, MPEC4839, MPEC4969, O32:H37 P4, P4-NR, and UVM2. **Orange:** The respective reference replicon from 1303 or ECC-1470. **Grey:** CDSs on the lagging and on the leading strand. **Blue or Brown:** Genomic islands/mobile elements or prophages. **(A)** Circular genome diagrams of all MAEC 1303 replicons: Chromosome, F-plasmid (p1303_109), circularized P1 bacteriophage (p1303_95), and small cryptic plasmid (p1303_5). **(B)** Circular genome diagrams of all MAEC ECC-1470 replicons: Chromosome and F-plasmid (pECC-1470_100).

Figure S6

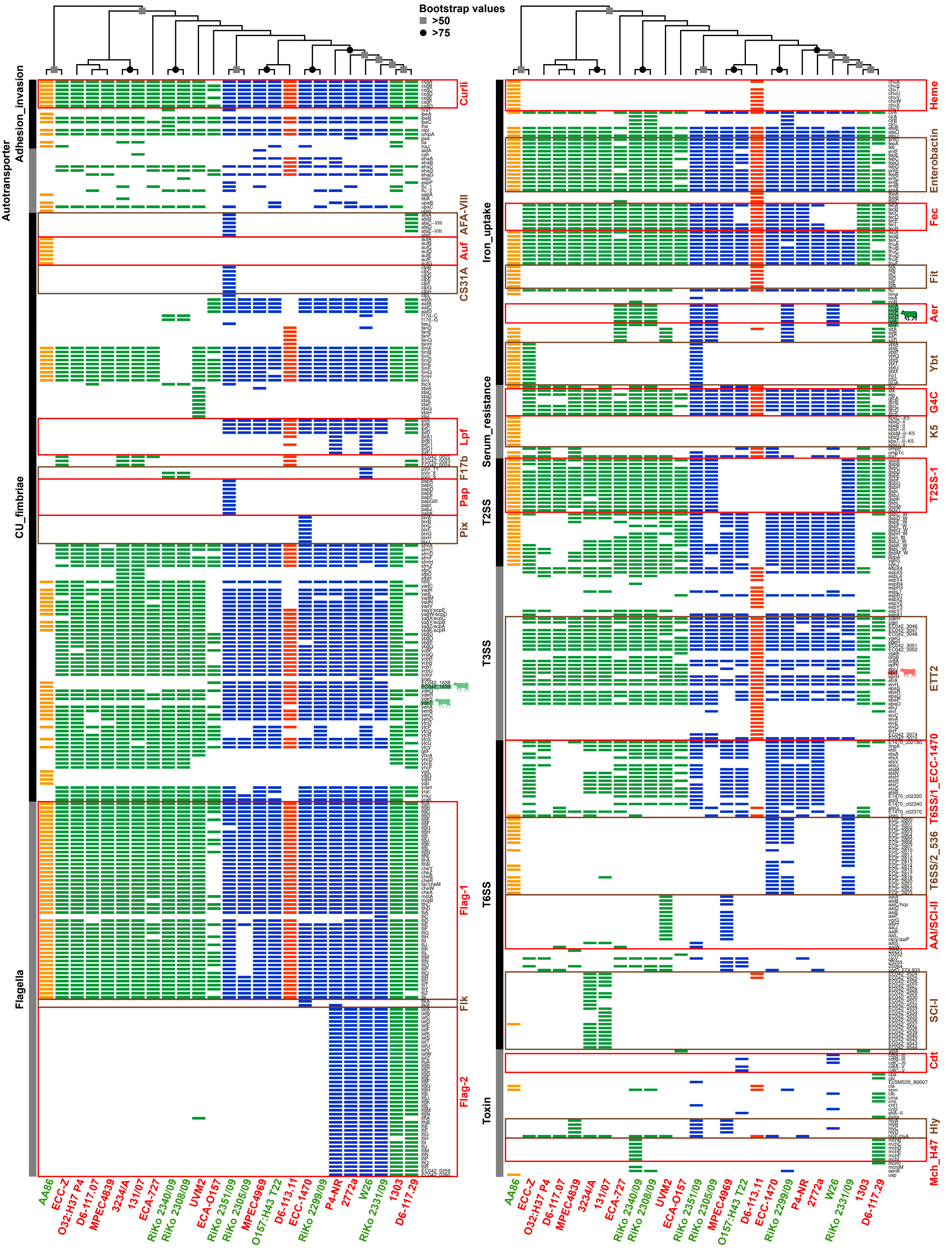

Figure S6. Heatmap of VF presence/absence. For the figure description see Figure 4. The only difference is the inclusion of virulence-associated gene names/locus tags.

Figure S7 A

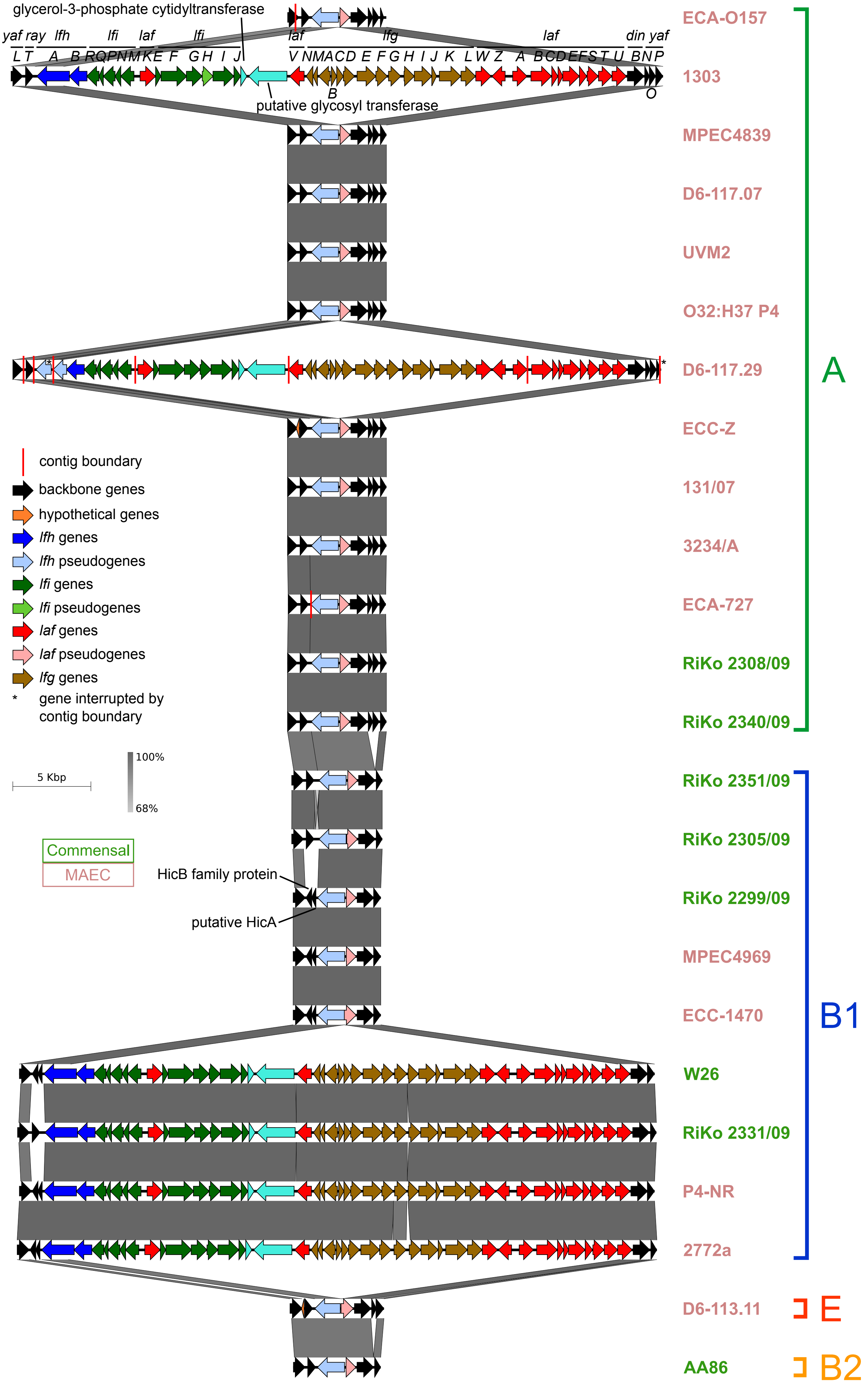
glycerol-3-phosphate cytidyltransferase
putative glycosyl transferase
HicB family protein
putative HicA
contig boundary
backbone genes
hypothetical genes
lfh genes
lfh pseudogenes
lfi genes
lfi pseudogenes
laf genes
laf pseudogenes
lfg genes
* gene interrupted by contig boundary
5 Kbp
100%
68%
Commensal
MAEC
ECA-O157
1303
MPEC4839
D6-117.07
UVM2
O32:H37 P4
D6-117.29
ECC-Z
131/07
3234/A
ECA-727
RiKo 2308/09
RiKo 2340/09
RiKo 2351/09
RiKo 2305/09
RiKo 2299/09
MPEC4969
ECC-1470
W26
RiKo 2331/09
P4-NR
2772a
D6-113.11
AA86
A
B1
E
B2

Figure S7 B

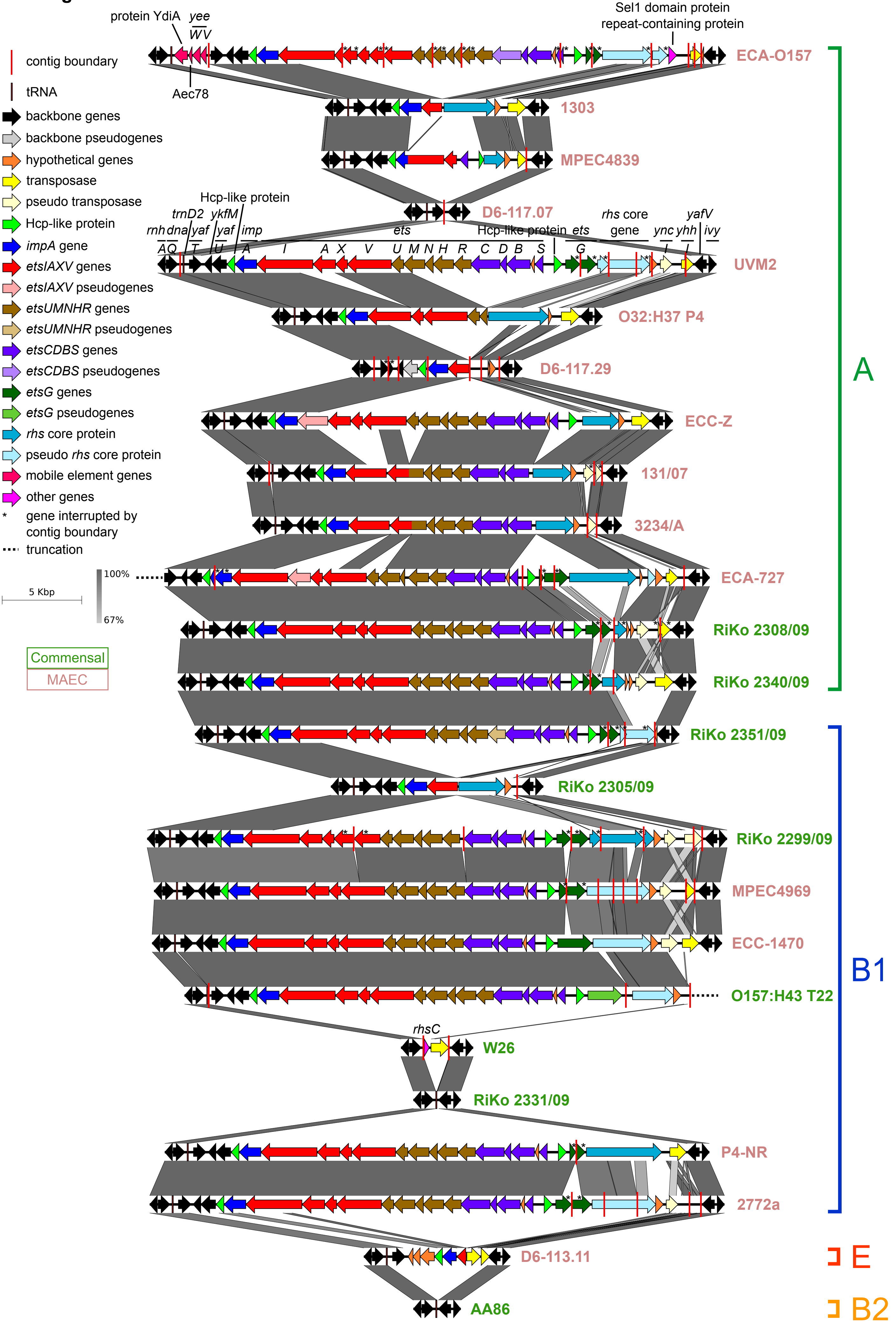

protein YdiA
yee
WV
Aec78
Sel1 domain protein
repeat-containing protein
contig boundary
tRNA
backbone genes
backbone pseudogenes
hypothetical genes
transposase
pseudo transposase
Hcp-like protein
impA gene
etsIAXV genes
etsIAXV pseudogenes
etsUMNHR genes
etsUMNHR pseudogenes
etsCDBS genes
etsCDBS pseudogenes
etsG genes
etsG pseudogenes
rhs core protein
pseudo rhs core protein
mobile element genes
other genes
* gene interrupted by contig boundary
truncation
100%
67%
5 Kbp
Commensal
MAEC
Hcp-like protein
trnD2 ykfM
rnh dna yaf yaf imp
AQ T U A
ets
I A X V U M N H R C D B S
Hcp-like protein ets
G
rhs core gene
ync yhh
I I
yafV
ivy
rhsC
ECA-O157
1303
MPEC4839
D6-117.07
UVM2
O32:H37 P4
D6-117.29
ECC-Z
131/07
3234/A
ECA-727
RiKo 2308/09
RiKo 2340/09
RiKo 2351/09
RiKo 2305/09
RiKo 2299/09
MPEC4969
ECC-1470
O157:H43 T22
W26
RiKo 2331/09
P4-NR
2772a
D6-113.11
AA86
A
B1
E
B2

Figure S7. Gene organization of the Flag-2 and MAEC ECC-1470 subtype i1 T6SS/1 gene clusters. Comparisons were done with Easyfig utilizing BLASTN+. Homologous regions are connected via grey vertices and colored by nucleotide identity. The genomes are ordered according to the WGA core genome phylogeny, ECOR phylogroups are indicated correspondingly. MAEC strain names are colored in light red and commensal strains in green. Gene names are indicated above genomes encoding for these. The respective contigs of the draft genomes containing the gene cluster were concatenated (contig boundaries are indicated by red vertical lines) and CDS spanning contig borders reannotated if needed (indicated by asterisks). Backbone genes not belonging to the putative VFs are colored black. Genes within each respective region have different colors (see the legend) to be able to evaluate their presence. Pseudogenes have a lighter color fill. **(A)** Flag-2 gene cluster comparison. *E. coli* strain O157:H43 T22 is omitted from the diagram because of a large deletion including also the Flag-2 flanking backbone genes. **(B)** MAEC ECC-1470 T6SS/1 gene organization comparison. The T6SS gene region of draft MAEC D6-117.29 could probably not be fully manually assembled, because of its high fragmentation. The *E. coli* strain O157:H43 T22 deletion encompasses also the T6SS downstream housekeeping genes, which is indicated by dots in the figure. Strain ECA-727 lacks the *yafT* to *impA* genes, because of a putative phage insertion in this region. This phage is not included in the figure and the truncation also indicated by dots.
