# Supplementary material for: No evidence for a bovine mastitis Escherichia coli pathotype
Source: BMC Genomics. 2017 May 8;18:359. doi: 10.1186/s12864-017-3739-x (PMC5422975; doi:10.1186/s12864-017-3739-x)
Supplement: Supplementary file 21 — Detailed Material & Methods description. (DOCX 108 kb) [file 12864_2017_3739_MOESM21_ESM.docx]

*Supplementary information*

No evidence for a bovine mastitis *Escherichia coli* pathotype

Andreas Leimbach^1,2,3^, Anja Poehlein^2^, John Vollmers^4^, Dennis Görlich^5^, Rolf Daniel^2^, Ulrich Dobrindt^1,3^

^1^ Institute of Hygiene, University of Münster, Münster, Germany

^2^ Department of Genomic and Applied Microbiology, Göttingen Genomics Laboratory, Institute of Microbiology and Genetics, Georg-August-University of Göttingen, Göttingen, Germany

^3^ Institute for Molecular Infection Biology, Julius-Maximilians-University of Würzburg, Würzburg, Germany

^4^ Leibniz Institute DSMZ, German Collection of Microorganisms and Cell Cultures, Braunschweig, Germany

^5^ Institute of Biostatistics and Clinical Research, University of Münster, Münster, Germany

## **Library preparation and sequencing**

Total DNA from overnight cultures for all strains was isolated with the MasterPure Complete DNA and RNA Purification Kit (Epicentre, Madison, WI, USA) according to the manufacturer's instructions. The strains with closed genomes, 1303 and ECC-1470, were sequenced as described by Leimbach and co-workers [[1](#_ENREF_1)]. In short, both genomes were first sequenced with the 454 Titanium FLX genome sequencer with GS20 chemistry (Roche Life Science, Mannheim, Germany) in a whole-genome shotgun approach to 27.8-fold and overall 13.4-fold coverage, respectively (384,786 reads and 143,474,880 bases for *E. coli* 1303, 129,126 reads and 39,329,989 bases for *E. coli* ECC-1470). Strain ECC-1470 was also sequenced with a 6-kb insert paired-end (PE) 454 library (155,130 reads and 26,495,179 bases).

These two strains were additionally and the draft strains [[2](#_ENREF_2)] solely sequenced with a 101-bp PE sequencing run on a HiScan SQ sequencer (Illumina, San Diego, CA, USA). For this purpose, sequencing libraries were prepared with Nextera XT chemistry. All Illumina raw reads were quality controlled with FastQC before and after trimming (v0.11.2; <http://www.bioinformatics.bbsrc.ac.uk/projects/fastqc>). Median insert sizes of the PE Illumina libraries were calculated with Picard's CollectInsertSizeMetrics (v1.124; <http://broadinstitute.github.io/picard>) after the raw reads were mapped onto the assembled contigs with Bowtie2 (v2.0.6) [[3](#_ENREF_3)] (see used options below) and the mappings sorted with SAMtools (v0.1.19) [[4](#_ENREF_4)]. Low quality 3’ end of reads and Illumina adapter contaminations (--stringency 2) were trimmed with cutadapt (v1.6) with a Q20 Phred score cutoff and a minimum read length of 20 bp [[5](#_ENREF_5)].

##

## **Assembly of the genomes**

Both 454 read sets for the genomes of *E. coli* 1303 and ECC_1470 were *de novo* assembled with Newbler (Roche) (v2.0.00.20 for *E. coli* 1303 and v2.3 for strain ECC-1470) [[6](#_ENREF_6)]. Additionally, these reads were assembled in a hybrid *de novo* approach in combination with the respective Illumina reads using MIRA (v3.4.0.1) [[7](#_ENREF_7)]. MIRA assembly of the corresponding reads with a 26x fold 454 and 70x fold Illumina coverage resulted in the following statistics for 1303: 98 contigs >= 500 bp and an N50 of 165,271 bp. ECC-1470 was initially assembled with reads of a 12x fold 454 and 75x fold Illumina coverage: 88 contigs >= 500 bp and an N50 of 194,065 bp. Afterwards, each 454 Newbler assembly was combined with the respective hybrid assembly in Gap4 (v4.11.2) of the Staden software package [[8](#_ENREF_8)]. The remaining gaps in the assembly were closed by primer walking via directed PCR and Sanger sequencing utilizing BigDye Terminator chemistry with ABI 3730 capillary sequencers. The sequences were processed with Pregap4 and loaded into the Gap4 databases. The closed genomes were edited to the “finished” standard [[9](#_ENREF_9)].

The Illumina reads from the draft *E. coli* genomes were each randomly subsampled to an approximate 70-fold coverage with seqtk (v1.0-r32; <https://github.com/lh3/seqtk>). Afterwards, the PE reads were *de novo* assembled with SPAdes (v3.1.1) with an iterative k-mer range of '-k 21,33,55,77' and option ‘--careful’ to reduce the number of mismatches and insertion/deletions [[10](#_ENREF_10)]. The following three steps were executed to check the assembled contigs: First, the reads used for the assemblies were mapped with Bowtie2 and its ‘--end-to-end’, ‘--very-fast’, and minimum (option ‘-I 0’) and maximum (‘-X 1000’) PE insert size options. The resulting SAM files were then sorted by coordinates and converted to BAM files with SAMtools to calculate mapping statistics with QualiMap (v2.0) [[11](#_ENREF_11)]. Only contigs >= 500 bp were retained, because smaller contigs often contain misassembled repeat sequences that cannot be resolved by the assembler. At last, the assembled contigs were ordered against the respective *E. coli* 1303 or *E. coli* ECC-1470 reference genomes, according to the ECOR phylogroup affiliation of the draft genomes. Contig ordering was done with ABACAS (v1.3.2) [[12](#_ENREF_12)] running NUCmer (v3.1) and with order_fastx (v0.1) [[13](#_ENREF_13)]. Assembly statistics were determined with QUAST (v3.2) [[14](#_ENREF_14)] using NUCmer from the MUMmer package (v3.23) [[15](#_ENREF_15)] for the 12 draft strains in this study and also for the 11 bovine-associated reference draft strains (with contigs >= 500 bp). All Sequence Read Archive (SRA) study accession numbers for the Illumina and 454 raw reads of the *E. coli* genomes of this study can be found in Additional file 5: Table S3. This file also includes the assembly statistics for all 23 bovine-associated *E. coli* draft genomes. The draft genomes of this study are in the “high-quality draft” standard [[9](#_ENREF_9)].

All genomes of this study were scanned with BLASTN+ (v2.2.28) [[16](#_ENREF_16)] for contamination with the Illumina phage PhiX spike-in control. Enterobacteria phage phiX174 genome (accession number: NC_001422.1) was used as query in the BLASTN+ runs.

##

## **Annotation of the genomes**

All strains of this study were initially automatically annotated with Prokka (v1.9) [[17](#_ENREF_17)] and the annotations subsequently supplemented with further databases. tRNAs were predicted with tRNAscan-SE (v1.3.1) [[18](#_ENREF_18)]. For the *E. coli* 1303 and ECC-1470 chromosomes *E. coli* K-12 MG1655 (accession number: NC_000913.3) and for their F plasmids (p1303_109 and pECC-1470_100) *E. coli* K-12 CR63 F plasmid (NC_002483.1) were used as references in Prokka (option ‘--proteins’). 1303 P1 phage plasmid (p1303_95) was annotated with enterobacteria phage P1 (NC_005856.1) as reference. These initial annotations were manually curated with the Swiss-Prot, TrEMBL [[19](#_ENREF_19)], IMG/ER [[20](#_ENREF_20)], and Ecocyc databases [[21](#_ENREF_21)]. Also, the Prodigal (v2.60) [[22](#_ENREF_22)] open reading frame (ORF) finding in Prokka was verified with a YACOP (v1) [[23](#_ENREF_23)] ORF finding. Subsequently, the two annotations were compared to the highly curated reference annotation of strain MG1655 using the Artemis Comparison Tool (ACT) (v12.1.1) [[24](#_ENREF_24)] with BLASTN+. With these comparisons manual curation was carried out with the tools Artemis (v15.1.1) [[25](#_ENREF_25)] and tbl2tab (v0.1) [[13](#_ENREF_13)]. Lastly, the annotations of *E. coli* strains 1303 and ECC-1470 were compared (ACT) and adapted to each other for a uniform annotation. The high quality annotation of the *E. coli* 1303 genome was then used as reference for the ECOR phylogroup A strains and the ECC-1470 genome annotation for the ECOR B1 strains during the Prokka annotation of the 12 draft genomes of this study. These annotations were further manually curated via ortholog/genome synteny analyses with the respective replicons of *E. coli* strains 1303 and ECC-1470 as references with Proteinortho (v5.11) [[26](#_ENREF_26), [27](#_ENREF_27)] (see options below) and po2anno (v0.2) [[13](#_ENREF_13)], ACT (v13.0.0) [[24](#_ENREF_24)] with BLASTN+, and cat_seq (v0.1) [[13](#_ENREF_13)]. At last, releases 1 (R1) and 2 (R2) of the Virulence Factors Database (VFDB) [[28](#_ENREF_28), [29](#_ENREF_29)], and the ResFinder (v2.1) [[30](#_ENREF_30)], VirulenceFinder (v1.2) [[31](#_ENREF_31)], and SerotypeFinder (v1.0) [[32](#_ENREF_32)] databases were used to refine the annotations with Artemis (v16.0.0) and tbl2tab (v0.2).

All eleven reference strains were also automatically reannotated with Prokka to have a uniform ORF-finding with Prodigal and facilitate comparative genomics. The draft genomes of D6-113.11 and D6-117_07.11 contain one contig each smaller than 200 bp. These two contigs were skipped by Prokka with the used option ‘--compliant’. The annotations of the references were shortly manually curated in the three putative virulence regions ETT2, Flag-2, and strain ECC-1470’s T6SS/1 by comparisons to the 1303 and ECC-1470 genomes as mentioned above. GENBANK files for these reannotations were created with NCBI’s tbl2asn (v24.3; <https://www.ncbi.nlm.nih.gov/genbank/tbl2asn2/>) with option ‘-V b’ and can be found in Additional file 22: Dataset S13 and Additional file 23: Dataset S14. For an overview of the annotations see the genome feature table created with genomes_feature_table (v0.5) [[13](#_ENREF_13)] (Additional file 1: Table S1). This table also includes the reference *E. coli* genomes for the phylogenetic analysis (see below), however their annotation features are listed as downloaded from NCBI.

## **Phylogenetic analysis**

For the phylogenetic analysis 39 additional reference *E. coli* strains (plus four *Shigella* spp. and one *Escherichia fergusonii* strain) were downloaded from NCBI with a wide variety of known pathotype and ECOR phylogroup affiliations. For the accession numbers see Additional file 1: Table S1. A whole genome nucleotide alignment (WGA) was done with the default parameter settings of Mugsy (v1.2.3) [[33](#_ENREF_33)] and the combined 68 *E. coli* genomes (including plasmids) with *E. fergusonii* as outgroup. This resulted in an original alignment length of 3,764,795 bp. The MAF alignment file was further processed to contain only locally colinear blocks without gaps present in all aligned genomes utilizing the software suite Phylomark (v1.3) [[34](#_ENREF_34)]. Phylomark in turn makes use of modules from Biopython (v1.63) [[35](#_ENREF_35)] and bx-python (v0.7.1; <https://github.com/bxlab/bx-python>), and as a final step runs mothur (v1.22.2) [[36](#_ENREF_36)]. After this treatment the resulting alignment length was 2,272,130 bp. The concatenated and filtered alignment was then subjected to RAxML (v8.1.22) [[37](#_ENREF_37)] to infer the best scoring ML phylogeny. RAxML was run with the GTRGAMMA generalized time-reversible (GTR) model of nucleotide evolution and GAMMA model of rate heterogeneity. 1,000 bootstrap resamplings were calculated with RAxML’s rapid bootstrapping algorithm (option ‘-f a’) for local support values. The resulting tree was visualized with Dendroscope (v3.4.4) [[38](#_ENREF_38)]. This phylogeny was used to classify the bovine-associated strains into ECOR phylogroups according to the included reference strains (with a known phylogeny) and monophyletic clades. The same procedure was followed including only the 25 bovine-associated *E. coli* strains. This resulted in a Mugsy alignment length of 4,312,845 bp and a filtered alignment length of 3,393,864 bp for RAxML. This tree was visualized with FigTree (v1.4.1; <http://tree.bio.ed.ac.uk/software/figtree/>) midpoint rooted.

Sequence types (STs) were assigned with ecoli_mlst (v0.3) [[13](#_ENREF_13)] according to the Achtman *E. coli* multi-locus sequence typing (MLST) scheme [[39](#_ENREF_39)] employing NUCmer with default parameters. Ambiguous allele numbers for strains ECA-O157, ECA-727, and O157:H7 EDL933 were resolved with BLASTN+ by choosing the sequence allele with the highest identity in the MLST database. PHYLOViZ (v1.1) [[40](#_ENREF_40)] was used to create a MST with the goeBURST algorithm [[41](#_ENREF_41)] to classify the STs into clonal complexes (CCs). CC numbers were allocated according to the Achtman *E. coli* MLST database. A CC is defined by STs that differ at maximal one locus/allele and are numbered by the founder of the CC, which is the ST with the highest number of neighboring single locus variants (SLVs). All allele, ST, and CC numbers can be found in Additional file 2: Table S2.

## **Detection of genomic islands and prophages, and generation of circular genome diagrams**

Because mobile genetic elements (MGEs) are prone to contain repetitive sequences, the short sequencing reads of most current high-throughput sequencing technologies cannot be unambiguously assembled in these regions [[42](#_ENREF_42)]. Additionally, automatic ORF prediction as well as annotation still remains a challenge in MGEs. Thus, we identified prophages and genomic islands (GIs) only for the two closed 1303 and ECC-1470 MAEC genomes. GIs were predicted with the three prediction methods of IslandViewer 3 [[43](#_ENREF_43)]: the two sequence composition methods SIGI-HMM [[44](#_ENREF_44)] and IslandPath-DIMOB, and the comparative genomic prediction method IslandPick [[45](#_ENREF_45)]. Only predicted GIs with a size greater than 8 kb were retained. Prophages were predicted with the PHAge Search Tool (PHAST) [[46](#_ENREF_46)]. PHAST also evaluates the completeness and potential viability of prophage regions by classifying them as “intact”, “questionable”, or “incomplete”. The GI and prophage predictions and their locations were evaluated manually by looking for mobility-associated genes, like integrases and transposons, toxin-antitoxin genes, restriction modification systems, and associated tRNAs using Artemis. The location, gene name (if available), locus tag, orientation, and product annotation was extracted for all genes included in the GI and prophage regions with Artemis.

Circular genome views were created with the BLAST Ring Image Generator (BRIG, v0.95) [[47](#_ENREF_47)] using BLASTP+ (v2.2.28) [[16](#_ENREF_16)] with a disabled low complexity filter (option ‘-seg no’) and upper/lower identity thresholds set to 90% and 70%, respectively. The location of the predicted GIs and prophages are visualized in these diagrams.

## **Identifying serotypes**

The SerotypeFinder (v1.0) database from the Center for Genomic Epidemiology was used to determine serotypes *in silico* [[32](#_ENREF_32)]. For some strains SerotypeFinder could not resolve the O- or H-antigen uniquely, in these cases both are listed.

## **Ortholog/paralog analysis**

Orthologous and paralogous proteins in all 25 bovine-associated genomes were identified with Proteinortho (v5.11) [26, [27](#_ENREF_27)] with a 1 x 10^-5^ E-value and 70% coverage/identity cutoffs. Proteinortho employs a bidirectional all-vs-all BLASTP+ (v2.2.29) approach using all predicted non-pseudo coding sequences, which were extracted from the genomes with cds_extractor (v0.7.1) and its option ‘-p’ [[13](#_ENREF_13)]. Additionally, Proteinortho’s ‘-synteny’ option was used to activate the PoFF module enabling the utilization of genome synteny for improving ortholog detection. GFF3 files for this purpose were created with bp_genbank2gff3.pl from the BioPerl script collection (v1.6.924; <https://github.com/bioperl/bioperl-live/tree/master/scripts/Bio-DB-GFF>) [[48](#_ENREF_48)]. Other non-default Proteinortho options used were a final local optimal Smith-Waterman alignment for BLASTP+ (‘-blastParameters=‘-use_sw_tback’’) recommended by Moreno-Hagelsieb and Latimer [[49](#_ENREF_49)] and Ward and Moreno-Hagelsieb [[50](#_ENREF_50)], ‘-selfblast’ for paralog detection, and ‘-singles’ to also report singletons. This resulted in a total number of 13,481 orthologous group (OGs) from the overall 116,535 CDSs in the bovine-associated strain panel.

To identify significant associations of OGs with pathotype (mastitis/commensal) or phylogroup (ECOR phylogroups A/B1), we employed a two-tailed Fisher’s Exact test provided in R (v3.2.5) and tested for OGs which are significantly (p<0.05) associated. Because phylogroups B2 and E contain only one genome each, they were omitted from Fisher’s exact test. These p-values were further evaluated with a Bonferroni correction. The negative base 10 logarithms of the Fisher’s exact test p-values were visualized as Manhattan plots with R package ggplot2 (v2.2.0) [[51](#_ENREF_51)]. The binary matrix for the OG presence/absence and the R script for the Fisher’s exact test are included in Additional file 4: Dataset S1.

Additionally, we considered OGs as pathotype- or phylogroup-enriched if they are minimally present in 70% of the genomes of one genome group (inclusion cutoff) and in maximally 30% of the genomes of all other groups (the other pathotype or phylogroups; exclusion cutoff) using po2group_stats (v0.1.1) [[13](#_ENREF_13)]. The 70%/30% inclusion/exclusion cutoffs amount to rounded 6/3 inclusion/exclusion genome cutoffs for the commensal isolates and 11/5 for the mastitis isolates. Similarly, the cutoffs in the phylogroups translate to rounded 9/4 genome inclusion/exclusion cutoffs for phylogroup A, 7/3 for B1, and 1/0 for the single genome groups B2 and E. According to these pathotype or phylogroup cutoffs, OGs are classified in “pathotype-/phylogroup-enriched”, “-absent”, “group soft core genome”, “underrepresented”, and “unspecific” (option ‘-u’) categories. OGs that are present >= the inclusion cutoff in the genomes of all groups are categorized in the “group soft core genome” category. The “underrepresented” category includes OGs present in <= genomes than the exclusion cutoff in all groups. Finally, OGs that are present in more genomes than the exclusion, but less than the inclusion cutoff in any group are categorized as “unspecific”. For each OG po2group_stats extracts the locus tag and annotation of one representative protein from one *E. coli* strain panel genome of the group (or in the case of paralogs several representative proteins).

The resulting Fisher’s exact test significant and pathotype-/phylogroup-enriched OG numbers from po2group_stats were visualized in venn diagrams (po2group_stats option ‘-p’) with the venn function of R package gplots (v3.0.1) [[52](#_ENREF_52)]. Additionally, singletons (option ‘-s’) were identified with po2group_stats.

In addition to the pathotype and phylogroup group soft core genomes calculated by po2group_stats, an “all-strain soft core genome” including all genomes with the 70% inclusion cutoff (18 out of 25 genomes) was determined. The all-strain soft core genome always includes more OGs than the pathotype/phylogroup group soft cores, because of the different number of groups the 70% inclusion cutoff is applied to. The difference originates from the inclusion of all OGs which are present in at least 70% of all genomes of each group in comparison to 70% of all genomes.

The resulting pathotype-enriched OGs were further evaluated by comparing their representative proteins to the representative proteins in the phylogroup-enriched categories and the all-strain soft core. The representative protein sequences were extracted from the respective GENBANK files with the locus tags included in the po2group_stats result files using cds_extractor (options ‘-p’ and ‘-l’). Subsequently, the prot_finder pipeline with BLASTP+ was used, as described in the virulence factore (VF) workflow, with the pathotype-enriched representative proteins as queries (option ‘-q’) and the phylogroup-enriched or all-strain/phylogroup soft core proteins as subjects (option ‘-s’).

Finally, a gene content tree was calculated with the Proteinortho presence/absence matrix of OGs (included in Additional file 4: Dataset S1). First, the matrix was converted to a binary matrix, transposed with transpose_matrix (v0.1) [[13](#_ENREF_13)], and then converted to FASTA format. This file was used to cluster the results by searching for the best scoring ML tree with RAxML’s (v8.0.26) BINGAMMA module (binary substitution model with GAMMA model of rate heterogeneity) and 1000 resamplings. The clustering tree was visualized midpoint rooted with Figtree.

## **Screening of the genomes for known virulence factors**

VF reference protein sequences were collected from the VFDB (R1 core dataset with experimentally validated VFs [[53](#_ENREF_53)], R2 comparative genomics dataset with intra-genera comparisons [[29](#_ENREF_29)], and R3 VF centric dataset with inter-genera comparisons [[28](#_ENREF_28)]) and reviewing the primary literature. For an overview of the VF panel see Additional file 12: Table S5. A focus was put on putative ExPEC VFs, because MAEC are considered to be ExPEC [[54](#_ENREF_54)]. The protein sequences of the VFs, as well as detailed information how the VF panel was collected, and the respective reference publications can be found in the GitHub repository <https://github.com/aleimba/ecoli_VF_collection> (v0.1) [[55](#_ENREF_55)].

The VF panel was used to assess the presence/absence of the 1,069 virulence-associated genes in the annotated bovine-associated strains with the prot_finder pipeline (v0.7.1) [[13](#_ENREF_13)] using BLASTP+ (v2.2.29). The following non-default options were used for the prot_finder pipeline: 1 x 10^-10^ E-value cutoff (‘-evalue 1e-10’), 70% query identity and coverage cutoffs (options ‘-i’ and ‘-cov_q’), and the best BLASTP hits option (‘-b’). This option includes only the hit with the highest identity for each subject CDS protein. A binary presence/absence matrix from these results was created with prot_binary_matrix (v0.6) and transpose_matrix (v0.1) [[13](#_ENREF_13)]. As with the gene content tree, a ML RAxML BINGAMMA search was done to cluster the results in the binary matrix with 1,000 resamplings. Additionally, the binary VF hit matrix was visualized with function heatmap.2 of the R package gplots and R package RColorBrewer (v1.1-2) [[56](#_ENREF_56)]. The aforementioned cladogram was attached to this heatmap with R package ape (v3.4) [[57](#_ENREF_57)]. The binary matrix, the cladogram NEWICK file, and the R script are included in Additional file 14: Dataset S7. The two resulting heatmaps were merged and edited in Inkscape.

A two-tailed Fisher’s exact test was used to identify VFs which are significantly (p < 0.05) associated with different pathotypes (mastitis/commensal) or phylogenetic groups (A/B1). P-values were also scrutinized with a Bonferroni correction. Manhattan plots were created with R package ggplot2. The R script for the Fisher’s exact tests and the Manhattan plots is in Additional file 4: Dataset S1. Again, inclusion and exclusion cutoffs were set to 70% and 30%, respectively, to identify VF associations with either pathotypes or phylogroups using binary_group_stats (v0.1) [[13](#_ENREF_13)]. Venn diagrams visualized the number of significant and pathotype-/phylogroup-enriched VF genes, as well as the group soft core VF sets. Also, an all-strain soft core VF set was calculated over the virulence-associated gene hits of all genomes with a 70% (18 genome) inclusion cutoff. Pathotype-enriched VF proteins were compared to phylogroup-enriched VF proteins for evaluation.

The same prot_finder pipeline and binary_groups_stats workflow was also used for two putative MAEC-specific regions in ECOR phylogroup A genomes [[58](#_ENREF_58)], which are not included in the VF panel. The first region is the biofilm-associated polysaccharide synthesis locus (*pgaABCD-ycdT-ymdE-ycdU*). The protein sequences from these genes were extracted from strain 1303 with cds_extractor (option ‘-l’). The locus tags are EC1303_c10400 to EC1303_c10440, EC1303_c10470, and EC1303_c10480. The second region encodes proteins involved in the phenylacetic acid degradation pathway (*feaRB-tynA-paaZABCDEFGHIJKXY*; MG1655 locus tags b1384 to b1400). The third region (the Fec uptake system, *fecIRABCDE*) is already included in the VF panel of this study. For this analysis the resulting binary BLASTP+ hit matrix was also tested with binary_groups_stats for pathotype association within the ECOR A and B1 phylogroups of the bovine-associated strain panel (with the 70% inclusion and 30% exclusion cutoffs). Associations were additionally controlled with Fisher’s exact test for significance.

## **Analysis of large structural putative virulence regions**

The composition of the large virulence regions ETT2, Flag-2, and the T6SS subtype i1 determinant of *E. coli* ECC-1470 was compared in more detail for the bovine-associated strain panel. To identify the corresponding contigs of the draft genomes the respective regions in *E. coli* strains 1303 and ECC-1470 were compared with ACT and BLASTN+ to the draft genomes. The identified draft contigs were optionally reversed with revcom_seq (v0.2), concatenated with cat_seq, and truncated with trunc_seq (v0.2) [[13](#_ENREF_13)] to include two flanking core genome genes. ORFs that spanned contig borders in the concatenated sequence files were manually elongated or added with Artemis, these genes are marked by asterisks ‘*’ in the figures. The genome comparison diagrams were created with Easyfig (v2.2.2) [[59](#_ENREF_59)] using BLASTN+ with a maximal E-value of 0.001 and the genomes ordered according to the WGA phylogeny.

The same workflow was done for the antimicrobial multidrug resistance element of 1303 (AMR-SSuT in GI4) in comparison to the *E. coli* SSuT-25 AMR-SSuT element [[60](#_ENREF_60)] (accession number: EF646764), the *E. coli* O157:H7 EC20020119 AMR-SSuT region (accession number: HQ018801) [[61](#_ENREF_61)], and transposon Tn*10* of *Shigella flexneri* 2b plasmid R100 (accession number: AP000342).

# **References**

1. Leimbach A, Poehlein A, Witten A, Scheutz F, Schukken Y, Daniel R et al. Complete Genome Sequences of *Escherichia coli* Strains 1303 and ECC-1470 Isolated from Bovine Mastitis. Genome Announc. 2015;3(2):e00182-15.

2. Leimbach A, Poehlein A, Witten A, Wellnitz O, Shpigel N, Petzl W et al. Whole-Genome Draft Sequences of Six Commensal Fecal and Six Mastitis-Associated *Escherichia coli* Strains of Bovine Origin. Genome Announc. 2016;4(4):e00753-16.

3. Langmead B, Salzberg SL. Fast gapped-read alignment with Bowtie 2. Nat Methods. 2012;9(4):357-9.

4. Li H, Handsaker B, Wysoker A, Fennell T, Ruan J, Homer N et al. The Sequence Alignment/Map format and SAMtools. Bioinformatics. 2009;25(16):2078-9.

5. Martin M. Cutadapt removes adapter sequences from high-throughput sequencing reads. EMBnetjournal. 2011;17:10.

6. Margulies M, Egholm M, Altman WE, Attiya S, Bader JS, Bemben LA et al. Genome sequencing in microfabricated high-density picolitre reactors. Nature. 2005;437(7057):376-80.

7. Chevreux B, Wetter T, Suhai S. 1999. Genome sequence assembly using trace signals and additional sequence information. Available from: <http://wwwbioinfode/isb/gcb99/talks/chevreux/mainhtml>.

8. Staden R, Beal KF, Bonfield JK. The Staden package, 1998. Methods Mol Biol. 2000;132:115-30.

9. Chain PS, Grafham DV, Fulton RS, Fitzgerald MG, Hostetler J, Muzny D et al. Genomics. Genome project standards in a new era of sequencing. Science. 2009;326(5950):236-7.

10. Bankevich A, Nurk S, Antipov D, Gurevich AA, Dvorkin M, Kulikov AS et al. SPAdes: a new genome assembly algorithm and its applications to single-cell sequencing. Journal of Computational Biology. 2012;19(5):455-77.

11. Okonechnikov K, Conesa A, Garcia-Alcalde F. Qualimap 2: advanced multi-sample quality control for high-throughput sequencing data. Bioinformatics. 2016;32(2):292-4.

12. Assefa S, Keane TM, Otto TD, Newbold C, Berriman M. ABACAS: algorithm-based automatic contiguation of assembled sequences. Bioinformatics. 2009;25(15):1968-9.

13. Leimbach A. bac-genomics-scripts: Bovine *E. coli* mastitis comparative genomics edition 2016.

14. Gurevich A, Saveliev V, Vyahhi N, Tesler G. QUAST: quality assessment tool for genome assemblies. Bioinformatics. 2013;29(8):1072-5.

15. Kurtz S, Phillippy A, Delcher AL, Smoot M, Shumway M, Antonescu C et al. Versatile and open software for comparing large genomes. Genome Biol. 2004;5(2):R12.

16. Camacho C, Coulouris G, Avagyan V, Ma N, Papadopoulos J, Bealer K et al. BLAST+: architecture and applications. BMC Bioinformatics. 2009;10:421.

17. Seemann T. Prokka: rapid prokaryotic genome annotation. Bioinformatics. 2014;30(14):2068-9.

18. Lowe TM, Eddy SR. tRNAscan-SE: a program for improved detection of transfer RNA genes in genomic sequence. Nucleic Acids Res. 1997;25(5):955-64.

19. UniProt Consortium. Activities at the Universal Protein Resource (UniProt). Nucleic Acids Res. 2014;42:D191-8.

20. Markowitz VM, Mavromatis K, Ivanova NN, Chen IM, Chu K, Kyrpides NC. IMG ER: a system for microbial genome annotation expert review and curation. Bioinformatics. 2009;25(17):2271-8.

21. Keseler IM, Mackie A, Peralta-Gil M, Santos-Zavaleta A, Gama-Castro S, Bonavides-Martinez C et al. EcoCyc: fusing model organism databases with systems biology. Nucleic Acids Res. 2013;41(Database issue):D605-12.

22. Hyatt D, Chen GL, Locascio PF, Land ML, Larimer FW, Hauser LJ. Prodigal: prokaryotic gene recognition and translation initiation site identification. BMC Bioinformatics. 2010;11:119.

23. Tech M, Merkl R. YACOP: Enhanced gene prediction obtained by a combination of existing methods. In Silico Biol. 2003;3(4):441-51.

24. Carver TJ, Rutherford KM, Berriman M, Rajandream MA, Barrell BG, Parkhill J. ACT: the Artemis Comparison Tool. Bioinformatics. 2005;21(16):3422-3.

25. Rutherford K, Parkhill J, Crook J, Horsnell T, Rice P, Rajandream MA et al. Artemis: sequence visualization and annotation. Bioinformatics. 2000;16(10):944-5.

26. Lechner M, Findeiss S, Steiner L, Marz M, Stadler PF, Prohaska SJ. Proteinortho: detection of (co-)orthologs in large-scale analysis. BMC Bioinformatics. 2011;12:124.

27. Lechner M, Hernandez-Rosales M, Doerr D, Wieseke N, Thevenin A, Stoye J et al. Orthology detection combining clustering and synteny for very large datasets. PLoS One. 2014;9(8):e105015.

28. Chen L, Xiong Z, Sun L, Yang J, Jin Q. VFDB 2012 update: toward the genetic diversity and molecular evolution of bacterial virulence factors. Nucleic Acids Res. 2012;40(Database issue):D641-5.

29. Yang J, Chen L, Sun L, Yu J, Jin Q. VFDB 2008 release: an enhanced web-based resource for comparative pathogenomics. Nucleic Acids Res. 2008;36:D539-42.

30. Zankari E, Hasman H, Cosentino S, Vestergaard M, Rasmussen S, Lund O et al. Identification of acquired antimicrobial resistance genes. J Antimicrob Chemother. 2012;67(11):2640-4.

31. Joensen KG, Scheutz F, Lund O, Hasman H, Kaas RS, Nielsen EM et al. Real-time whole-genome sequencing for routine typing, surveillance, and outbreak detection of verotoxigenic *Escherichia coli*. J Clin Microbiol. 2014;52(5):1501-10.

32. Joensen KG, Tetzschner AM, Iguchi A, Aarestrup FM, Scheutz F. Rapid and Easy *In Silico* Serotyping of *Escherichia coli* Isolates by Use of Whole-Genome Sequencing Data. J Clin Microbiol. 2015;53(8):2410-26.

33. Angiuoli SV, Salzberg SL. Mugsy: fast multiple alignment of closely related whole genomes. Bioinformatics. 2011;27(3):334-42.

34. Sahl JW, Matalka MN, Rasko DA. Phylomark, a tool to identify conserved phylogenetic markers from whole-genome alignments. Appl Environ Microbiol. 2012;78(14):4884-92.

35. Cock PJ, Antao T, Chang JT, Chapman BA, Cox CJ, Dalke A et al. Biopython: freely available Python tools for computational molecular biology and bioinformatics. Bioinformatics. 2009;25(11):1422-3.

36. Schloss PD, Westcott SL, Ryabin T, Hall JR, Hartmann M, Hollister EB et al. Introducing mothur: open-source, platform-independent, community-supported software for describing and comparing microbial communities. Appl Environ Microbiol. 2009;75(23):7537-41.

37. Stamatakis A. RAxML version 8: a tool for phylogenetic analysis and post-analysis of large phylogenies. Bioinformatics. 2014;30(9):1312-3.

38. Huson DH, Scornavacca C. Dendroscope 3: an interactive tool for rooted phylogenetic trees and networks. Syst Biol. 2012;61(6):1061-7.

39. Wirth T, Falush D, Lan R, Colles F, Mensa P, Wieler LH et al. Sex and virulence in *Escherichia coli*: an evolutionary perspective. Mol Microbiol. 2006;60(5):1136-51.

40. Francisco AP, Vaz C, Monteiro PT, Melo-Cristino J, Ramirez M, Carrico JA. PHYLOViZ: phylogenetic inference and data visualization for sequence based typing methods. BMC Bioinformatics. 2012;13:87.

41. Francisco AP, Bugalho M, Ramirez M, Carrico JA. Global optimal eBURST analysis of multilocus typing data using a graphic matroid approach. BMC Bioinformatics. 2009;10:152.

42. Treangen TJ, Salzberg SL. Repetitive DNA and next-generation sequencing: computational challenges and solutions. Nature Reviews Genetics. 2011;13(1):36-46.

43. Dhillon BK, Laird MR, Shay JA, Winsor GL, Lo R, Nizam F et al. IslandViewer 3: more flexible, interactive genomic island discovery, visualization and analysis. Nucleic Acids Res. 2015;43(W1):W104-8.

44. Waack S, Keller O, Asper R, Brodag T, Damm C, Fricke WF et al. Score-based prediction of genomic islands in prokaryotic genomes using hidden Markov models. BMC Bioinformatics. 2006;7:142.

45. Langille MG, Hsiao WW, Brinkman FS. Evaluation of genomic island predictors using a comparative genomics approach. BMC Bioinformatics. 2008;9:329.

46. Zhou Y, Liang Y, Lynch KH, Dennis JJ, Wishart DS. PHAST: a fast phage search tool. Nucleic Acids Res. 2011;39(Web Server issue):W347-52.

47. Alikhan NF, Petty NK, Ben Zakour NL, Beatson SA. BLAST Ring Image Generator (BRIG): simple prokaryote genome comparisons. BMC Genomics. 2011;12:402.

48. Stajich JE, Block D, Boulez K, Brenner SE, Chervitz SA, Dagdigian C et al. The Bioperl toolkit: Perl modules for the life sciences. Genome Res. 2002;12(10):1611-8.

49. Moreno-Hagelsieb G, Latimer K. Choosing BLAST options for better detection of orthologs as reciprocal best hits. Bioinformatics. 2008;24(3):319-24.

50. Ward N, Moreno-Hagelsieb G. Quickly finding orthologs as reciprocal best hits with BLAT, LAST, and UBLAST: how much do we miss? PLoS One. 2014;9(7):e101850.

51. Wickham H. Elegant Graphics for Data Analysis. New York: Springer; 2009.

52. Warnes GR, Bolker B, Bonebakker L, Gentleman R, Liaw WHA, Lumley T et al. gplots: various R programming tools for plotting data. 2016.

53. Chen L, Yang J, Yu J, Yao Z, Sun L, Shen Y et al. VFDB: a reference database for bacterial virulence factors. Nucleic Acids Res. 2005;33:D325-8.

54. Shpigel NY, Elazar S, Rosenshine I. Mammary pathogenic *Escherichia coli*. Curr Opin Microbiol. 2008;11(1):60-5.

55. Leimbach A. ecoli_VF_collection: v0.1. Zenodo. 2016.

56. Neuwirth E. RColorBrewer: ColorBrewer palettes. 2014.

57. Popescu AA, Huber KT, Paradis E. ape 3.0: New tools for distance-based phylogenetics and evolutionary analysis in R. Bioinformatics. 2012;28(11):1536-7.

58. Goldstone RJ, Harris S, Smith DG. Genomic content typifying a prevalent clade of bovine mastitis-associated *Escherichia coli*. Sci Rep. 2016;6:30115.

59. Sullivan MJ, Petty NK, Beatson SA. Easyfig: a genome comparison visualizer. Bioinformatics. 2011;27(7):1009-10.

60. Khachatryan AR, Besser TE, Call DR. The streptomycin-sulfadiazine-tetracycline antimicrobial resistance element of calf-adapted *Escherichia coli* is widely distributed among isolates from Washington state cattle. Appl Environ Microbiol. 2008;74(2):391-5.

61. Ziebell K, Johnson RP, Kropinski AM, Reid-Smith R, Ahmed R, Gannon VP et al. Gene cluster conferring streptomycin, sulfonamide, and tetracycline resistance in *Escherichia coli* O157:H7 phage types 23, 45, and 67. Appl Environ Microbiol. 2011;77(5):1900-3.
